# Mice with Deficient BK Channel Function Show Impaired Prepulse Inhibition and Spatial Learning, but Normal Working and Spatial Reference Memory

**DOI:** 10.1371/journal.pone.0081270

**Published:** 2013-11-26

**Authors:** Marei Typlt, Magdalena Mirkowski, Erin Azzopardi, Lukas Ruettiger, Peter Ruth, Susanne Schmid

**Affiliations:** 1 Department of Anatomy and Cell Biology, Schulich School of Medicine and Dentistry, University of Western Ontario, London, Ontario, Canada; 2 Hearing Research Center, UniversitätTübingen, Tübingen, Germany; 3 Pharmakologie, Toxikologie und Klinische Pharmazie, Institut für Pharmazie, UniversitätTübingen, Tübingen, Germany; Universidad de Castilla-La Mancha, Spain

## Abstract

Genetic variations in the large-conductance, voltage- and calcium activated potassium channels (BK channels) have been recently implicated in mental retardation, autism and schizophrenia which all come along with severe cognitive impairments. In the present study we investigate the effects of functional BK channel deletion on cognition using a genetic mouse model with a knock-out of the gene for the pore forming α-subunit of the channel. We tested the F1 generation of a hybrid SV129/C57BL6 mouse line in which the *slo1* gene was deleted in both parent strains.

We first evaluated hearing and motor function to establish the suitability of this model for cognitive testing. Auditory brain stem responses to click stimuli showed no threshold differences between knockout mice and their wild-type littermates. Despite of muscular tremor, reduced grip force, and impaired gait, knockout mice exhibited normal locomotion. These findings allowed for testing of sensorimotor gating using the acoustic startle reflex, as well as of working memory, spatial learning and memory in the Y-maze and the Morris water maze, respectively.

Prepulse inhibition on the first day of testing was normal, but the knockout mice did not improve over the days of testing as their wild-type littermates did. Spontaneous alternation in the y-maze was normal as well, suggesting that the BK channel knock-out does not impair working memory. In the Morris water maze knock-out mice showed significantly slower acquisition of the task, but normal memory once the task was learned. Thus, we propose a crucial role of the BK channels in learning, but not in memory storage or recollection.

## Introduction

The large-conductance, voltage- and calcium-activated potassium channels (BK channels) are expressed throughout the mammalian nervous system [1]–[3] and can be found at neuronal soma and processes, as well as on presynaptic terminals [1], [4], [5]. Activated by membrane depolarization and/or micromolar concentrations of intracellular calcium, BK channels drive the membrane potential towards the potassium equilibrium potential and therefore re- and hyperpolarize the neuron [6]–[8]. By limiting the duration of action potentials, they regulate the general excitability of neurons [9]–[11], as well as the transmitter release at presynaptic terminals [8], [12]–[15].

Over the last several years evidence has accumulated that alterations in the expression and function of BK channels are involved in a number of mental disorders. So far, functional alterations of these channels have been linked to mental retardation [16], [17] , autism [18], and schizophrenia (for review see [19]) which all involve sensorimotor, as well as cognitive impairments.

In order to explore the role of BK channels in cognitive processes we tested a mouse model lacking functional BK channels. Previous studies found that BK channel knock-out mice with a 129SVJ inbred background have impaired hearing [20]–[22] and motor function [23], which would impact most of the standard behavioral tests. However, another study in mice with a FVB/NJ inbred background suggests strain specific differences in the hearing ability of BK channel knock-out mice [24]. We here used the F1 generation of a hybrid SV129/C57BL6 background. Several studies indicate that using a hybrid or mixed background in F1 or F2 generation is most effective to contrast the gene knock-out to natural occurring variations in behaviour due to genetic background and environmental factors [25]–[27].

After confirming normal hearing and locomotion, we first tested sensorimotor gating. Sensorimotor gating is a pre-attentive process that filters sensory information for salient stimuli within the environmental noise. In all patient groups linked with an alteration in BK channel expression disruptions in sensorimotor gating have been described (fragile X syndrome: [28], autism: [29], [30], and schizophrenia: [31]–[33]). Therefore, we hypothesized that sensorimotor gating will be impaired in the BK channel knockout mice. We further tested the BK knockout mice in the Y-maze to assess their working memory, as well as in the Morris water maze to assess hippocampus dependent learning and memory. Since BK channels are abundantly expressed in the hippocampus [2], [3], [34], we expected to find deficits specifically in these tasks.

## Methods

### Animals and animal care

We used mice of the F1 generation of a hybrid SV129/C57BL6 line with deficient BK channel function. BK channel function was abolished by deleting the *slo1* gene which encodes the pore forming channel protein (α-subunit; for details see [23]). Heterozygous C57BL6 mice were paired with heterozygous SV129 mice. Exclusively mice of the respective F1 generation were tested in order to avoid effects of inbreeding. Also, preliminary data had suggested that these hybrid mice do not show hearing deficits as previously described in other genetic models with impaired BK channel function [20], [21]. In total, we tested 20 wild-type (WT; 10/10 male/female), 17 heterozygote (BKα^+/−^; 10/7), and 19 homozygote knock-out mice (BKα^−/−^; 11/8), but different, randomly selected, subsets of these animals were used for the single tests. The hearing test was performed on a separate set of animals (for details see respective method sections). The animals were litter- and/or age matched. We only tested mice at ages from 3 to 7 months to avoid effects of aging.

All mice were generated and genotyped at the Pharmaceutical Institute, University of Tübingen, Germany. In order to ensure proper tracking each mouse got an individual ear-tag when collecting the DNA sample for the genotyping. The mice were shipped to Canada at the age of 1.5-3 months and subsequently allowed to acclimate for 2 weeks before behavioural testing started. Mice were group housed with mixed genetic background within groups, with a 12 hour light-dark cycle and with ad libitum food and water. Testing only occurred during the light cycle from 7am to 7pm. If animals were tested repeatedly over several days, the time of testing for each animal was kept constant.

All procedures were in accordance with the ethical guidelines of the Canadian Council on Animal Care (CCAC) and approved by the University of Western Ontario Animal Use Subcommittee.

### Hearing measurements/auditory brainstem responses (ABR)

Hearing measurements were conducted at the University of Tübingen, Germany, and were in accordance with institutional and national guidelines following approval by the University of Tübingen, Veterinary Care Unit, and the Animal Care and Ethics Committee of the regional board of the Federal State Government of Baden-Württemberg, Germany.

Auditory brainstem responses (ABR) were recorded from 10 WT (5/5 male/female) and 8 BKα^−/−^ mice (6/2), as described previously [35]. Each mouse was tested at several time points between the age of 4–15 weeks (9±3.5 weeks) to evaluate the development of the hearing thresholds. Briefly, recordings were performed while mice were under ketamine (75 mg/kg body weight) and xylazine (5 mg/kg body weight) anesthesia. ABRs to free field click (100 µs), and pure tone (3 ms, 1 ms ramp) acoustic stimuli were recorded using sub-dermal silver wire electrodes at the ear (active), the vertex (reference) and the back (ground) of the animals. Signals were amplified (50-100-fold), band-pass filtered (200 Hz high-pass and 5 kHz low-pass), and averaged for 64-256 repetitions at each sound pressure level (SPL) presented (0 -100 dB in steps of 5 dB). Hearing threshold was defined as the lowest SPL that produced a potential visually distinct from background noise.

### Grip force test

In order to assess muscular strength, the grip force test was used. The front limb strength of 16 animals of each genotype (WT: 9 males/ 7 females, BKα^+/−^: 10/6, BKα^−/−^: 9/7) was measured in 5 trials per animal. The animals were between 7 and 9 months (34±2 weeks) old at the time of testing. A force gauge (Grip Strength Meter, Columbus Instruments, Columbus, Ohio, USA) was connected to a metal grid which allowed the animal to hold on. The animal was held by the base of its tail and lowered towards the grid until it gripped it with both front limbs. Then the animal was gently pulled back until it lost the grip. Between trials the animals were allowed to recover in their home cages for about 10 minutes. For subsequent analyses the results from the 5 trials were averaged for each animal.

### Cat walk

To evaluate gait 11 WT (6 males/5 females), 11 BKα^+/−^ (7/4), and 10 BKα^−/−^ mice (7/3) aged 7 to 9 month (33±2 weeks) were tested on the CatWalk (Noldus Information Technology Inc, Leesburg, VA, USA). Animals traversed a transparent walkway through which they were video-taped. Animals were allowed to walk back and forth until a minimum of 5 consecutive steps could be recorded. Using the CatWalk™software (Version 7.0, Noldus Information Technology Inc, Leesburg, VA, USA) the paw prints of the animals were tracked. We analyzed stride length and paw print area of each individual paw, as well as inter-limb coordination using the regularity index (RI) as introduced by Hamers et al. [36]. The regularity index grades the inter-limb coordination as the ratio between the number of normal step sequence patterns and the total number of paw placements. A RI of 100% indicates perfect inter-limb coordination.

### Open field locomotor activity

General locomotor activity was measured in 16 mice of each genotype (WT: 9 males/ 7 females, BKα^+/−^: 10/6, BKα^−/−^: 9/7) at the age of 6 to 9 month (30±2.5 weeks). Each animal was placed in a square open field box (Versamax Animal Activity Monitor, AccuScan Instruments, Colombus, OH, USA) for 2 hours. During the whole time of testing the room was moderately lit (200 lux). Using the VersMax™ software (AccuScan Instruments) we analyzed the distance traveled during the 2 hours, the number of rearing movements, as well as the time spent in the center versus the time spent at the margins as a measure of anxiety.

### Acoustic startle reflex/prepulse inhibition (PPI)

Prepulse inhibition of the acoustic startle reflex was used to measure sensorimotor gating. 18 WT (10 males/8 females), 17 BKα^+/−^ (10/7), and 19 BKα^−/−^ mice (9/10) were tested as described previously [37], [38]. Only animals aged 4-6 months (21.5±2 weeks) were tested to avoid possible effects of age induced hearing loss. Testing was conducted in sound attenuated startle boxes from MED Associates (MED-ASR-PRO1, St Albans, VT, USA) using the associated software for stimulus presentation and recordings (Startle Reflex, Version 6.0, MED Associates, Inc.).

Sound was presented by two loud speakers in each startle box. The first speaker presented a constant background noise (65 dB SPL white noise), whereas the second speaker presented the startle and prepulse stimuli. The sound was calibrated with the sound pressure level measurement package provided by MED Associates (ANL-929A-PC) placing the microphone into the animal holder at approximately the same distance to the speaker as the animals’ head during the test sessions. To record the startle response, the animals were placed into a tube like plastic animal holder which was then mounted on top of a piezo driven transducer which translated vertical movements into a voltage signal. The voltage signal in response to a given stimulus was amplified, digitalized and stored on a computer. We started the recording 50 ms before any stimulus was given and recorded for a total of 500 ms per stimulus.

Before the actual testing the animals were acclimatized to the startle boxes for 3 days to reduce anxiety. During these 5 minute long acclimation phases the background noise was presented. On the third day, these 5 minutes were followed by a short input/output (I/O) test to determine the appropriate gain setting for the transducer signal for each individual animal. The I/O test consisted of 12 acoustic stimuli with increasing intensity (65–120 dB SPL, 20 ms duration). Once set, the gain was kept constant for all recordings of a given animal. In order to reduce variability we tested each animal on 5 consecutive days using the following protocol.

Animals were first acclimatized to the startle boxes as during the acclimation days, and then habituated to the startle stimulus to avoid habituation effects during the prepulse testing. For habituation, the startle stimulus (20 ms, 105 dB SPL white noise) was presented 100 times with varying inter-trial intervals (10–20 seconds). The prepulse subsequent test consisted of 7 different trial conditions which were each presented 10 times in a pseudo-randomized order and at varying inter-trial intervals (10–20 seconds). The trial conditions included startle alone trials to determine the baseline startle, and trials with one of two types of acoustic prepulses (4 ms, 75 and 85 dB SPL, white noise) leading the startle pulse by 10, 30, or 100 ms. Unfortunately, animals sometimes startled to the prepulse of 85 dB SPL, thus we omitted these trials from further analysis, since effects of muscle fatigue could not be excluded.

For each trial the startle amplitude was calculated as the difference between the maximum positive and negative displacement of the transducer induced by the startling animal. Prepulse inhibition was calculated for each animal and each trial condition as PPI (%)  =  (1- average startle amplitude to pulse with prepulse/average startle amplitude to pulse only)*100.

### Y-maze

In order to asses working memory and exploration behavior 15 mice of each genotype (WT: 9 males/ 6 females, BKα^+/−^: 10/5, BKα^−/−^: 7/8) aged between 8 and 10 month (36.5±2 weeks) were tested in the Y-maze. The custom-made maze consisted of three white plastic arms separated by a 120° angle. Each arm was 30 cm long, 10 cm wide, and restricted by 20 cm high walls. The room in which the test was performed was moderately lit (200 lux). The animals were placed in the center of the maze and were allowed to explore freely for 5 minutes, during which the number and order of arm entries was recorded. An arm entry was defined as the animal placing all four paws into a given arm. Spontaneous alternation was calculated as the ratio of number of triads (sequence of 3 consecutive arm entries visiting all 3 arms) and total arm entries.

The strength of the spontaneous alteration task is that it requires no prior training, nor food or water restriction. The animals naturally tend to explore new environments and therefore prefer to investigate a new arm of the maze rather than returning to the previously explored one. In order to achieve this, the animals need to recall which arm they visited last (for review see: [39]–[41]).

### Water maze

To assess spatial reference learning and memory we tested 14 mice of each genotype (WT: 9 males/ 4 females, BKα^+/−^: 10/4, BKα^−/−^: 7/7) in the Morris water maze. The animals were between 10 and 12 month (46±3 weeks) old at the time of testing. We used a circular pool with a diameter of 1.5 meters and 60 cm high walls. The water was 45 cm deep and at room temperature (24°C). The pool was divided into equally sized quadrants in each of which a squared platform (10×10 cm) could be positioned in the middle. The platform was transparent and about 1.5 cm below the water surface to avoid that the animals can see it. Permanent visual cues on the room walls allowed for spatial orientation. A camera mounted above the pool recorded every trial and animals were tracked using the ANY-maze™ software (Version 4.98, Stoelting Co, Wood Dale, IL, USA)

Each animal underwent four training days with four trials a day (10-15 minutes inter-trial interval). The platform position was assigned randomly, but was counterbalanced for the genotypes. Once assigned, the platform position for a given animal was not changed between trials and days. However, the starting position of the animal was changed between every trial (first trial randomized, for the following trials the position moved clockwise between the quadrants). If the mouse did not find the platform within 90 seconds it was guided to the platform on the first trial. The animals were allowed to sit on the platform for 10 to 15 second and were then moved back into their home cages. For every trial we analyzed the time it took the animal to reach the platform. On the fifth day we ran a 60 second probe trial without the platform and recorded the time the animals spent in the quadrant where the platform previously was hidden.

### Statistics

Data analyses and graphical display were done with Microsoft Excel (Version 14.0.6129.5000, Microsoft Corp.), Graphpad (for graphical display, Prism 6.01, Graphpad Software, LaJolla, CA, USA) and SPSS (for statistical analysis, Version 20.0.0, IBM Corp.). Data are expressed as mean ± S.E. We also displayed the standard error in the graphs as measure of precision of the sample mean.

Depending on the paradigm, a 2-Way-ANOVA, a MANOVA, or a repeated measurement ANOVA with genotype and gender as between-subjects factors was performed to compare groups.

For repeated measurement ANOVAs the Mauchly test was used to judge if the data violated the sphericity assumption. In case of a violation the degrees of freedom were corrected using the Greenhous-Geisser (if ε < .75) or the Huynh-Feldt method (if ε > .75). For post hoc analyses the Sidak t-test was performed. Differences were considered statistically significant when p-values were smaller than 0.05. In the figures significance levels between genotypes were indicated as followed: * *p* < .05, **p < .01, *** *p* < .001.

## Results

We tested the F1 generation of homozygous BKα knockout mice (BKα^−/−^), as well as their heterozygous (BKα^+/−^), and wildtype (WT) littermates for their hearing ability and motor function. Subsequently, we assessed their cognitive functions using a variety of tests.

All mice had a normal development of weight, from 15 g body weight at the age of 4 weeks up to 30 g at 15 weeks. There was no significant difference in the absolute weight and weight gain between the WT and the BKα^−/−^ mice. We also did not find a gender effect for the given age range tested (see Fig. S1).

### Hearing

**Figure 1:**
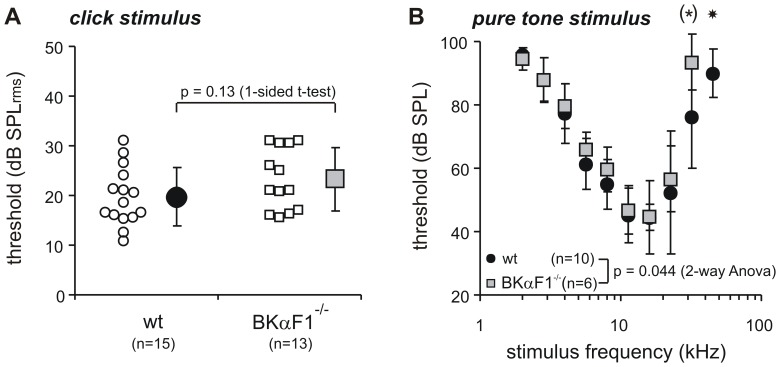
Hearing thresholds measured with auditory brainstem response. (**A**) Click thresholds of WT and BKα^−/−^ mice. Mean and S.D. (filled symbols). Single animal thresholds are shown as smaller, open symbols beside either group mean. The difference in threshold (3 dB) was statistically not significant. (**B**) Pure tone thresholds (mean and S.D.) as function of stimulus frequency for WT (black circles) and BKα^−/−^ mice (grey squares). Threshold curves were just significantly different, based on significant elevated thresholds at 32 kHz (*:p’ = 0.048, 1-sided t-test without alpha correction) and a drop out of threshold for BKα-/- mice at 45 kHz (star: p = 0.0262, Fisher Exact Probability Test).

Hearing function was measured by ABR thresholds. Thresholds for click evoked ABRs were not statistically different for WT (19.8±5.8 dB SPL) and BKα^−/−^ mice (23.3±6.2 dB SPL, *p*  =  0.13, Fig.1A); and within the tested age range (4 to 15 weeks) the ABR thresholds did not change significantly (see Fig. S2). Consistent with the thresholds for the (broadband) click stimulus, the thresholds for frequency specific pure-tone stimuli were similar for WT and BKα^−/−^ mice over the low and middle frequency range of hearing (4- 22 kHz, Fig.1B). At frequencies of 32 kHz and above the hearing thresholds of the BKα^−/−^ mice were significantly elevated and not even detectable at 45 kHz (2-way ANOVA: p = 0.044, 45 kHz: p = 0.0262). Despite this high frequency hearing loss, a progression of hearing thresholds upon aging could not be found within the frequency range of best hearing (8–16 kHz, also addressed with the click stimulus, see Fig.S2).

Thus, we concluded that cognitive tests that require normal hearing function can be performed with this animal model, as long as testing occurs in the low and/or mid frequency range.

### Motor Functions

BK channels are abundantly expressed in the cerebellum and other motor areas in the brain. Therefore, deficient BK channel function might affect motor function. Since almost all cognitive tests rely on motor behavior and/or locomotion, we next tested these in our mice.

**Figure 2:**
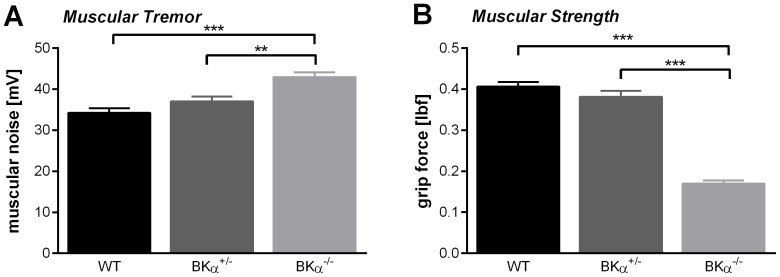
Muscle functions. (**A**) Muscular tremor was measured employing data recorded during the startle testing. During periods of no acoustic stimulation the noise of the recorded signal was significantly higher in the BKα^−/−^ mice. (**B**) In addition to the tremor the BKα^−/−^ mice also showed significant reduced muscle strength judging by their front limb grip force.

All BKα^−/−^ mice had a noticeable muscle tremor, whereas their BKα^+/−^ and WT littermates moved normally. Apart from the just visual evidence, we were able to detect and measure this tremor during the startle testing. A two-way ANOVA revealed that the voltage signal on the movement sensitive platform during the periods before the acoustic stimulation was significantly different for the genotypes (Fig.2A, *F*(2,50)  =  393.24, *p* < .001, no gender effect, nor genotype x gender interaction). The BKα^−/−^ mice had a significant higher noise (43.0±1.2 mV) compared to the WT (34.3±1.1 mV, *p* < .001) and BKα^+/−^ mice (37.07±1.17 mV, *p*  =  .003). This increased noise was not due to initiated movements of the mice during this brief period since that would have resulted in much larger signal amplitudes. All the recordings were visually inspected to exclude trials with spontaneous movement. Thus, the increased noise seems to reflect the muscular tremor seen in the BKα^−/−^ mice.

In order to assess the muscular strength of the mice we used the grip force test (Fig.2B). A two-way ANOVA reported a significant difference between the genotypes (*F*(2,42)  =  119.03, *p* < .001). BKα^+/−^ mice (0.38±0.01 lbf) showed similar strength as WT mice (0.41±0.01 lbf, *p*  =  .361), whereas BKα^−/−^ mice showed significant lower muscular strength compared to both, WT (*p* < .001) and BKα^+/−^ mice (*p* < .001). With a grip force of on average 0.17±0.01 lbf they had less than half the muscle strength of their WT littermates. There was no gender effect, nor a genotype x gender interaction for the grip force.

**Figure 3:**
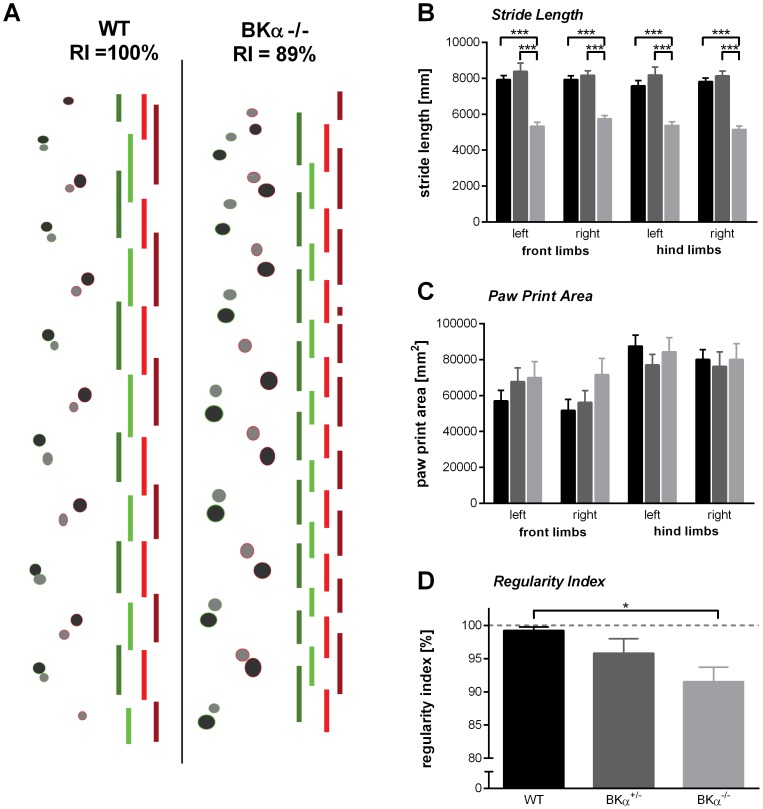
Gait strait analysis. (**A**) Examples of the paw print and their timing for WT (left) and BKα^−/−^ mice (right). The bars indicating the timing of each single paw on the floor (from left to right: left hind limb, left front limb, right front limb, right hind limb). (**B**) Stride length for all paws is reduced in the BKα^−/−^ mice, but (**C**) no significant differences were found for the paw print areas. (**D**) The inter-limb coordination as measured with the regularity index in reduced in BKα^−/−^ mice. The dashed line shows a regularity index of 100% indicating perfect inter-limb coordination.

In order to evaluate the ability of the mice to walk properly, they were tested on the cat walk. We analyzed stride length and paw print area of each individual paw, as well as inter-limb coordination. An example of paw prints and the respective timing of the paws is given in Fig.3A for both WT and BKα^−/−^ mice. A MANOVA on all parameters captured from the catwalk, reported a highly significant difference between genotypes (*λ_Wilks_*  =  .082, *F*(18,36)  =  5.00, *p* < .001, no gender effect and no genotype x gender interaction).

Most prominent was the difference between the genotypes in the stride length (Fig.3B). The effect was consistent throughout all four limbs, but we will describe this effect here using the left front limb as an example. For the left limb stride length a follow-up two-way ANOVA reported significant differences for the genotype (*F*(2,26)  =  32.6, *p* < .001). The stride length of the BKα^−/−^ mice was about 30% shorter than that of WT and BKα^+/−^ mice, with an average stride length of 5407±150 mm as opposed to 7815±220 mm (*p* < .001) and 8219±330 mm *(p* < 0.001), respectively. In contrast, the paw print area did not differ between genotypes for any of the paws (Fig.3C).

In order to grade the inter-limb coordination, the regularity index (RI) was calculated. Whereas 81% (9/11) of the WT animals were rated as having a perfect RI, only 45% (5/11) of the BKα^+/−^ mice and only 27% (3/11) of BKα^−/−^ mice were rated perfect. Indeed, the average regularity index for the genotypes differed significantly (*F*(2,26)  =  4.35, *p*  =  .023, Fig.3D). With a RI_BKα-/-_  =  91.5±2.2% the BKα^−/−^ mice showed significantly less regularity in their walking pattern than the WT mice which had an average RI_WT_  =  99.3±0.5% (*p*  =  .037). With a RI  =  95.84±2.2% the BKα^+/−^ mice showed intermediate inter-limb coordination, but it was not significantly different to either the WT (*p*  =  .416), or the BKα^−/−^ mice (*p*  =  .507).

**Figure 4:**
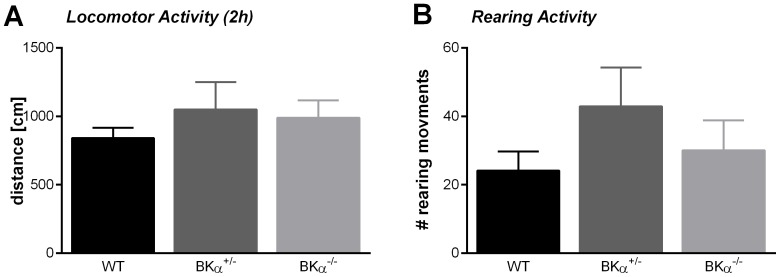
Locomotion. (**A**) BKα^−/−^ mice show normal locomotion in the open field box as well as (**B**) normal rearing activity.

Despite the BKα^−/−^ mice showing tremor, reduced muscle strength, and gait disturbances, they exhibited normal locomotor behavior (Fig.4). A two hour test in the open field box yielded no significant differences between the genotypes (MANOVA, *λ_Wilks_*  =  .928, *F*(6,80)  =  0.64, *p*  =  .801, no gender effect, nor genotype x gender interaction). On average the BKα^−/−^ mice traveled the same distance (989±127 cm) with the same speed (9.8±0.7 cm/s) and showed similar rearing activity (30.1±8.8) as their WT (distance  =  840±76 cm, speed  =  9.0±0.2 cm/s, rearings  =  24.1±5.6,) and BKα^+/−^ litter mates (distance  =  1050±200 cm, speed  =  9.4±0.3 cm/s, rearings  =  42.9±11.3). There was also no significant difference between genotypes for the respective parameters within the first 5 minutes of the open field test. To further assess locomotion, we also measured the swim speed of the mice in the Morris water maze. We could not detect any differences in swim speed between genotypes (*F*(2,36)  =  1.20, *p  = * .314, no gender effect, nor genotype x gender interaction, data not shown).

We conclude that BKα^−/−^ mice show normal locomotion but disrupted fine motor coordination. Therefore, cognitive tests that rely on normal locomotion can be performed with this animal model, as long as fine motor coordination is not required.

### Sensorimotor gating

We originally tested prepulse inhibition over five days to reduce variability in the data. Interestingly, the repeated measurements ANOVA (inter-stimulus interval x day) did not only report a genotype effect (*F*(2,48)  =  6.04, *p  = * .005, no gender effect, no genotype x gender interaction), but also a significant effect for the day of testing (*F*(3.922,188.275)  =  7.198, *p <* .001) and a day x genotype interaction (*F*(7.845,188.275)  =  2.155, *p*  =  .034). Therefore, we analyzed the first day of testing separately and then additionally analyzed the development of prepulse inhibition across the 5 days of testing.

**Figure 5:**
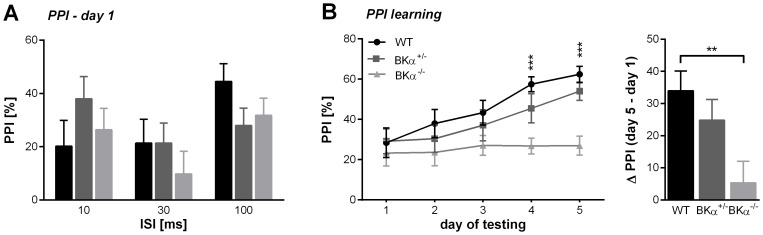
Prepulse inhibition (PPI) of the acoustic startle response. (**A**) On the first day of testing no significant differences in prepulse inhibition were found between genotypes for all tested inter-stimulus intervals. (**B**) Across days the WT and BKα^+/−^ mice showed improvement in PPI whereas the BKα^−/−^ mice did not. The average PPI for all tested ISI is shown, since no effect of ISI was reported by the repeated measurements ANOVA. The right panel shows the average difference in PPI between the fifth and first day of testing. Differences were calculated for each single animal and then averaged for genotypes.

For the first day of testing the repeated measurements ANOVA (inter-stimulus interval) on prepulse inhibition did not reveal any significant differences between the genotypes (*F*(2,50)  =  0.338, *p  = * .715), nor an effect of gender, or a genotype x gender interaction (Fig.5A), suggesting that the deficit in BK channel function does not impair prepulse inhibition. However, we did find that the prepulse inhibition in WT and BKα^+/−^ improved over the days of testing, whereas in BKα^−/−^ mice it stayed constant (Fig.5B). Since the best prepulse inhibition was typically seen on the fifth day of testing, we computed the difference between the performance on day five and one for each animal to judge their improvement of PPI over days. The ANOVA confirmed that in the BKα^−/−^ mice prepulse inhibition improved significantly less than in their WT littermates (*F*(2,49)  =  5.05, *p  = * .010, no gender effect, no genotype x gender interaction). While in the WT mice prepulse inhibition on day five was 34.0±6.1% higher, it was only improved by 5.3±6.7% in the BKα^−/−^ mice.

### Spontaneous Alternation

**Figure 6:**
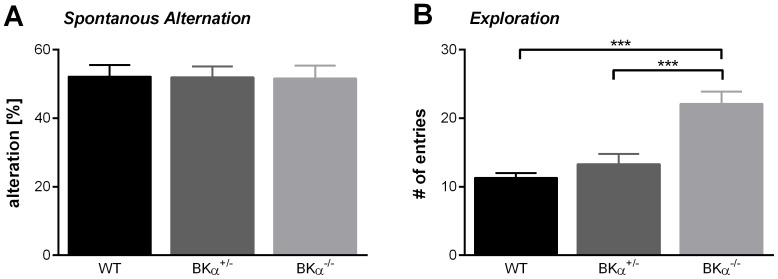
Spontaneous alternation in the Y-maze. ( **A**) Spontaneous alternations are presented as percent of total arm entries. There was no difference between the genotypes. (**B**) The number of total arm entries is significantly increased in the BKα^−/−^ mice.

We next tested our animals for spatial working memory using the spontaneous alternation task in the Y-maze. A two-way ANOVA reported no significant differences between the genotypes (*F*(2,39)  =  0.04, *p  = * .960, Fig.6A). All genotypes showed on average 52% alternation, suggesting that there is no impairment in spatial working memory in the BKα^−/−^ mice. However, note that there was a significant difference in the number of total arm entries between the genotypes (*F*(2,39)  =  17.13, *p <* .001, Fig.6B). The BKα^−/−^ mice showed twice as many arm entries (22.1±1.8) as the WT (11.3±0.7, *p* < .001) and BKα^+/−^ mice (13.3±1.5, *p* < .001), and therefore spent less time in a arm per entry. It could be observed that they did not explore the arms completely. This possibly indicates a different exploratory behaviour strategy. There was no gender effect, nor a genotype x gender interaction for spontaneous alterations or number of arm entries.

### Spatial learning and memory

**Figure 7:**
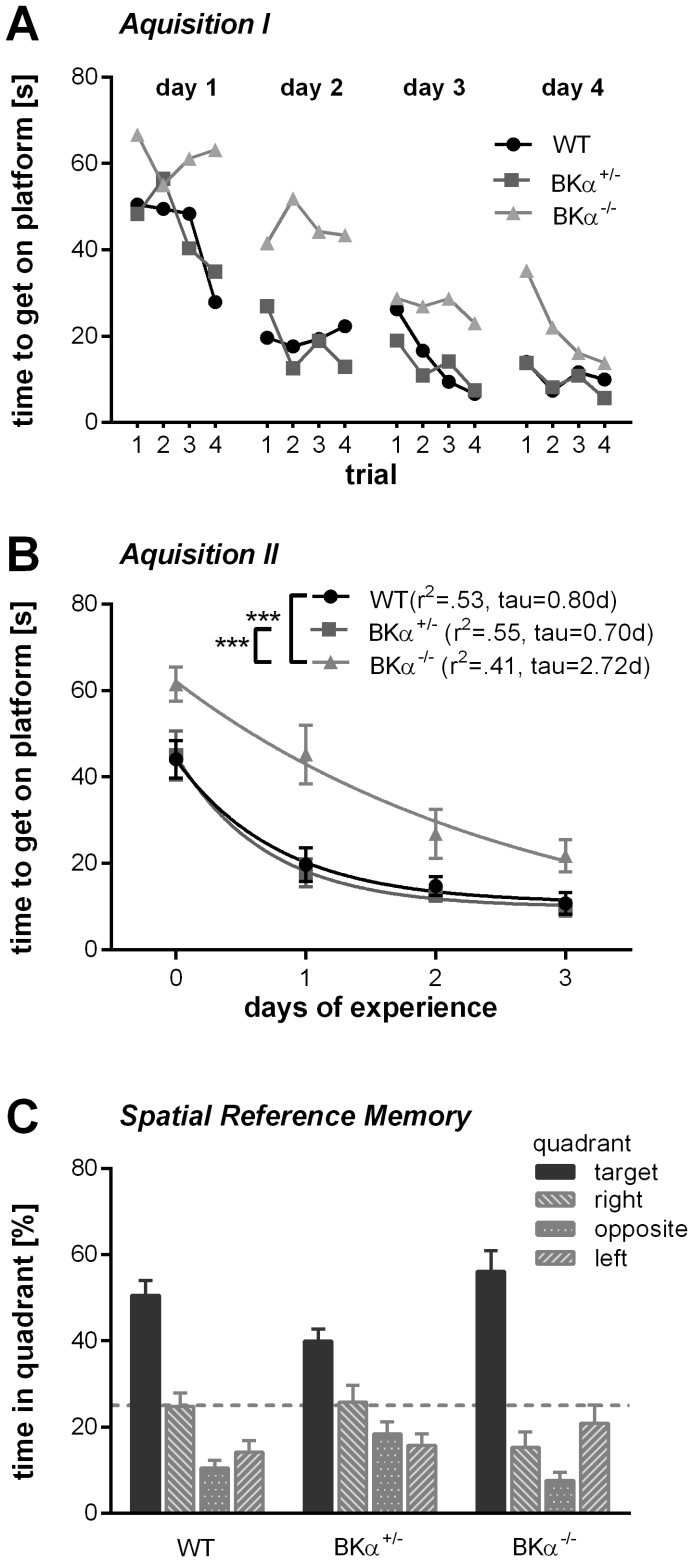
Spatial learning and memory in the Morris water maze. (**A**) The average escape latency improved across the training trails in all genotypes, but especially on days one to three, the BKα^−/−^ mice improve less within the training sessions. Note that on the last training trail on day 4 they caught up and performed as well as the WT mice. (**B**) Escape latency as a function of days of experience with the task. The average escape latency for each day was fitted with a one component exponential function for each genotype and time constants were derived from the resulting functions. The BKα^−/−^ mice have a longer time constant then the WT and the BKα^+/−^ mice. (**C**) Performance on the probe day. All mice spend a significant higher amount of time in the quadrant where previously the platform was hidden (target, solid dark grey) than in any other quadrant. The dashed line indicates 25% chance criterion.

Finally, we tested the animals for spatial reference learning and memory in the Morris water maze. The animals underwent four training days with four trials a day. As mentioned previously, there was no difference in the swim speed for the genotypes. However, repeated measurements ANOVA (day x trials) for the time to reach the platform reported significant differences between genotypes (*F*(2,36)  =  11.33, *p <* .001, no gender effect, nor genotype x gender interaction). The ANOVA also found a main effect for the day (*F*(3,108)  =  57.65, *p <* .001) and for the trial (*F*(3,108)  =  5.306, *p  = * .002), but there was no interaction with genotype for neither of them, nor a day x trial x genotype interaction. The BKα^−/−^ mice took longer to reach the platform throughout all trials (Fig.7A, B). On average, it took the BKα^−/−^ mice 38.8±2.8 seconds to get to the platform, while the WT and BKα^+/−^ mice reached the platform in 22 seconds (WT  =  22.1±2.9 s, *p <* .001, BKα^−/−^  =  22.6±3.1 s, *p <* .001).

When analyzing the performance within days, the BKα^−/−^ mice show less improvement over the trials, especially on days one to three. However, they did improve over the days; it just took them longer to learn the task than their WT and BKα^+/−^ littermates. Indeed, the exponential fits to the data yielded a longer time constant for the BKα^−/−^ mice (τ  =  2.72 days) compared to the WT (τ  =  0.80 days) and BKα^+/−^ mice data (τ  =  0.70 days, Fig.7B). Eventually they do seem to catch up on day four where they did perform as well as WT and BKα^+/−^ mice in the last trial.

Once the task was learned, the BKα^−/−^ mice did remember the localization of the platform as well as their WT and BKα^+/−^ littermates. On the probe day all mice spent a significantly higher amount of time in the quadrant where previously the platform was hidden compared to the other quadrants (Fig.7C). A MANOVA on the time in the target quadrant, as well as the time in the neighboring and opposite quadrants did not find a genotype effect (*λ_Wilks_*  =  .698, *F*(6,66)  =  2.17, *p*  =  .057), nor a gender effect, or a genotype x gender interaction. The WT and BKα^−/−^ mice spent an equal amount of time in the quadrant where previously the platform was hidden (51.6±4.2% and 56.2±3.9% respectively). Thus, we conclude that the BKα^−/−^ mice do not have spatial memory deficits.

In summary, the present study suggests that a knockout of the α-subunit of the BK channel results in deficits in learning. The spatial working memory and the spatial reference memory seem not to be affected.

## Discussion

In the present study we used for the first time a genetic model of BK channel deficiency to test the role of BK channels in cognition. We used mice with a hybrid SV129/C57BL6 background which are not hearing impaired and show normal locomotion. We found that the ablation of functional BK channels disrupts prepulse inhibition learning and slows down the acquisition of new tasks, but does not affect memory.

### Auditory processing and motor function in BK channel knockout mice

We first verified that our hybrid SV129/C57BL6 BKα^−/−^ mice could perform all tasks required for the cognitive tests that we employed. One of the major concerns was their hearing. Rüttiger et al. [20] showed that BK channels are critical for the survival of high frequency responding outer hair cells in the basal cochlea and that a deletion of the pore forming α-subunit of the BK channel leads to progressive hearing loss with age starting around eight weeks at which the high frequency range seems to be especially affected. In contrast, Pyott et al. [24] did not find any age-dependent changes in auditory thresholds. Whereas Rüttiger et al. [20] tested a 129SVJ inbred mouse line, Pyott et al. [24] worked with a FVB/NJ inbred mice. Thus, the hearing phenotype of BKα subunit deficient mice seems to be strain specific. Further, Pyott et al. [24] suggested that the BK channels are only required under extreme levels of hair cell activity where they serve to protect normal hearing.

In the present study we used a hybrid SV129/C57BL6 mouse line and we could not find any age depending reduction of hearing thresholds for the click stimuli, which were later used in the acoustic startle reflex experiments. However, we still see an effect of the BK channel function loss on the high frequency range hearing thresholds. This high frequency hearing loss should not affect the acoustic startle reflex experiments since the click stimuli used there only include low to mid range frequencies. BK channels can not only alter hearing thresholds, but also temporal coding of sounds [21], [22] which could prevent the detection of the short (4 ms) prepulse in the acoustic startle experiments. However, Kurt et al. [22] showed that mice with a conditional BK channel knockout for the inner hair cells surprisingly have improved discrimination of temporally modulated sounds. Thus, the BK channel knockout is unlikely to have affected the detection of the prepulse in our experiments.

Another major concern with general BK channel knockout mice is their motor function. BK channels are abundantly expressed in the cerebellum [1], [3], [23] and loss of BK channel function has been shown to lead to cerebellar ataxia with intentional tremor, abnormal gait, and motor coordination [23]. We mainly confirmed most of these findings in the mouse model used here. However, despite tremor, impaired muscle strength, and gait disturbances the mice exhibited normal locomotor behavior, for both walking and swimming, making it suitable for cognitive tests involving locomotion, but not fine motor coordination.

### BK channels in sensorimotor gating

BK channels deficiency has been implicated in mental retardation/ fragile X syndrome [16], [17] , autism [18], and schizophrenia (for review see [19]). Impaired prepulse inhibition is one common symptom of these mental disorders (mental retardation: [28], autism: [29], [30], schizophrenia: [31]–[33]). Thus, we expected to find deficits in prepulse inhibition in the BK channel knockout mice. However, prepulse inhibition in our BK channel knockout mice was not significantly different to that of their wild-type littermates on the first day of testing. Prepulse inhibition was quite low for both the wild-type and the knockout mice with only about 20-30%, which has been reported before for different mice strains (compare [42], [43]).

Interestingly, we found a deficit in prepulse inhibition learning. Wild-type mice showed an increase in prepulse inhibition across the five days of testing to an average of around 60% whereas the knockout mice did not substantially improve. This effect cannot be attributed to differences in the baseline startle, since both wild-type and knockout mice had similar baseline startle amplitudes. A floor effect in the knockout mice can be further excluded because the absolute startle amplitude in the wild-type mice was smaller than in the knockout mice in the presence of a prepulse, and all startle amplitudes were at least twice the noise level and well detectable (data not shown). Experience-dependent increase in prepulse inhibition has been described before and has been attributed to an associative learning process which links the prepulse with the startle stimulus in a timely manner [42], [43]. However, it is unclear where and how exactly this effect is mediated. Our data suggests a critical role for the BK channels in mechanisms mediating prepulse inhibition learning.

### BK channels in learning and memory

Another common symptom of the mental disorders linked with BK channel deficiency is cognitive disruption. BK channels are expressed almost throughout the entire central nervous system, but the expression is particularly high in the hippocampus [2], [3], [34]. Thus, it is likely that hippocampus dependent tasks are impaired if BK channel function is disrupted. Indeed, it took BK channel knockout mice longer to learn the Morris water maze task. Matthews and Disterhoft [44] reported a comparable effect on trace eyeblink conditioning when blocking BK channels in the hippocampus with intercranial injections of paxilline. The animals that had received drug injections learned the association of the auditory stimulus with the air puff to the eye considerably slower than the control animals. Sausbier et al. [23] also reported slower acquisition of the conditioned eyeblink reflex in global BK channel knockout mice (129SVJ inbred), but they attributed it to a cerebellar learning deficit. In line with these findings is also the deficit in prepulse inhibition learning in the BK channel knockout mice which is attributed to an associated learning process as discussed above. Taken together, these studies indicate a crucial role for the BK channels in synaptic mechanisms underlying learning. This might be an evolutionary highly conserved mechanism, as a calcium dependent potassium current also has been linked to associative learning in molluscs [45].

How exactly the BK channels are involved in learning is not fully understood yet. BK channels mediate the fast after-hyperpolarization and thus regulate intrinsic neuronal excitability [7], [10], [46], [47]. Blocking the BK channels with either paxilline or iberiotoxin increases the firing rate to step currents in *in vitro* whole-cell recordings [10], as well as the *in vivo* spontaneous firing rate of hippocampal CA1 [48]. Additionally, BK channels have been shown to regulate transmitter release at the presynaptic terminals [8], [12], [13]. Blocking the BK channels enhances the probability of transmitter release at CA3-CA3 synapses in the hippocampus [12]. Together those effects lead to an increased neuronal noise which reduces the dynamic range for neurons to code information on a firing rate base in BK channel deficient systems. However, BK channels are expressed abundantly in the central nervous system, it is therefore possible that other structures beside the hippocampus are involved in mediating the described effects. In particular, the substantia nigra, the habenula, and striatum also have been shown to be involved in associative learning and they express BK channels [49], [50].

Interestingly, memory, once it is established seems not to be affected by the BK channel knock-out. Both, working memory (which was measured with the y-maze spontaneous alternation task), as well as the spatial reference memory (which was measured on the probe day in the Morris water maze), were comparable between the knock-out and the wild-type mice.

## Conclusion

The present study shows that BK channel knock-out mice do not improve prepulse inhibition of startle over days and are slower in learning the Morris water maze task, suggesting a critical role for the BK channels in mechanisms underlying associative learning. BK channels may therefore become an increasingly interesting target for the development of new drugs for enhancing cognitive function.

## Supporting Information

Figure S1 Weight gain in males (blue) versus females (red) and wild-types (dots) versus knock-out (squares) mice. No difference in body weight gain was observed between any of these groups during 4 to 15 weeks of age.
(TIF)

Figure S2 ABR hearing thresholds for WT (black circles) and BKα-/- mice (grey squares) for click stimuli did not change significantly with age. Animals measured on more than one time point are connected with lines.
(TIF)
