# Supplementary figures and images for: Mice with Deficient BK Channel Function Show Impaired Prepulse Inhibition and Spatial Learning, but Normal Working and Spatial Reference Memory
Source: PLoS One. 2013 Nov 26;8(11):e81270. doi: 10.1371/journal.pone.0081270 (PMC3841135; doi:10.1371/journal.pone.0081270)

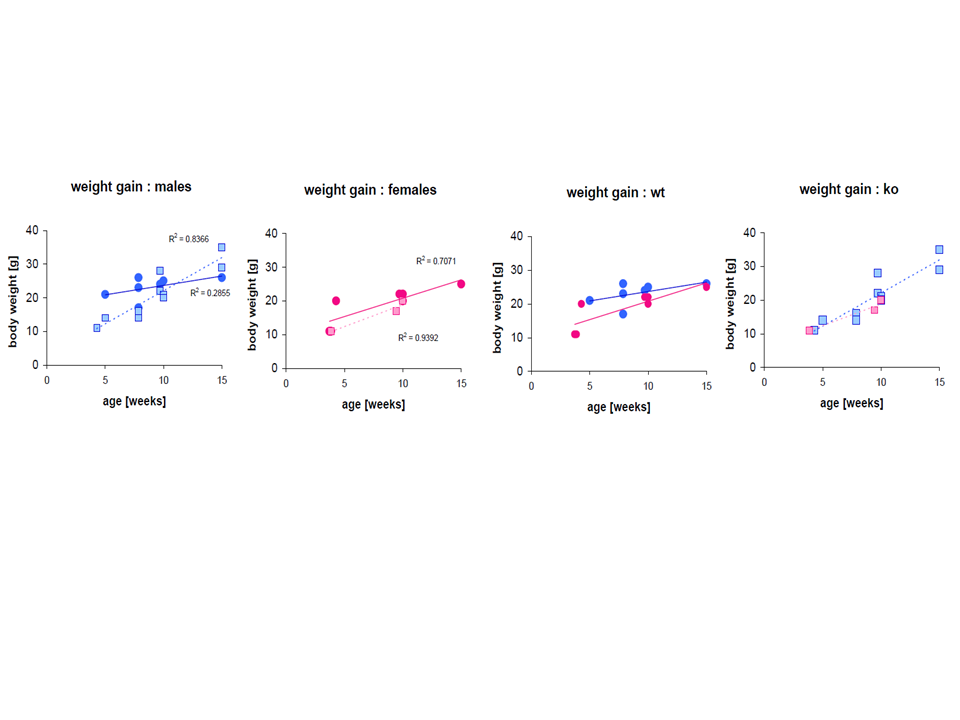

Supplement: Figure S1 — Weight gain in males (blue) versus females (red) and wild-types (dots) versus knock-out (squares) mice. No difference in body weight gain was observed between any of these groups during 4 to 15 weeks of age. (TIF) [file pone.0081270.s001.tif]

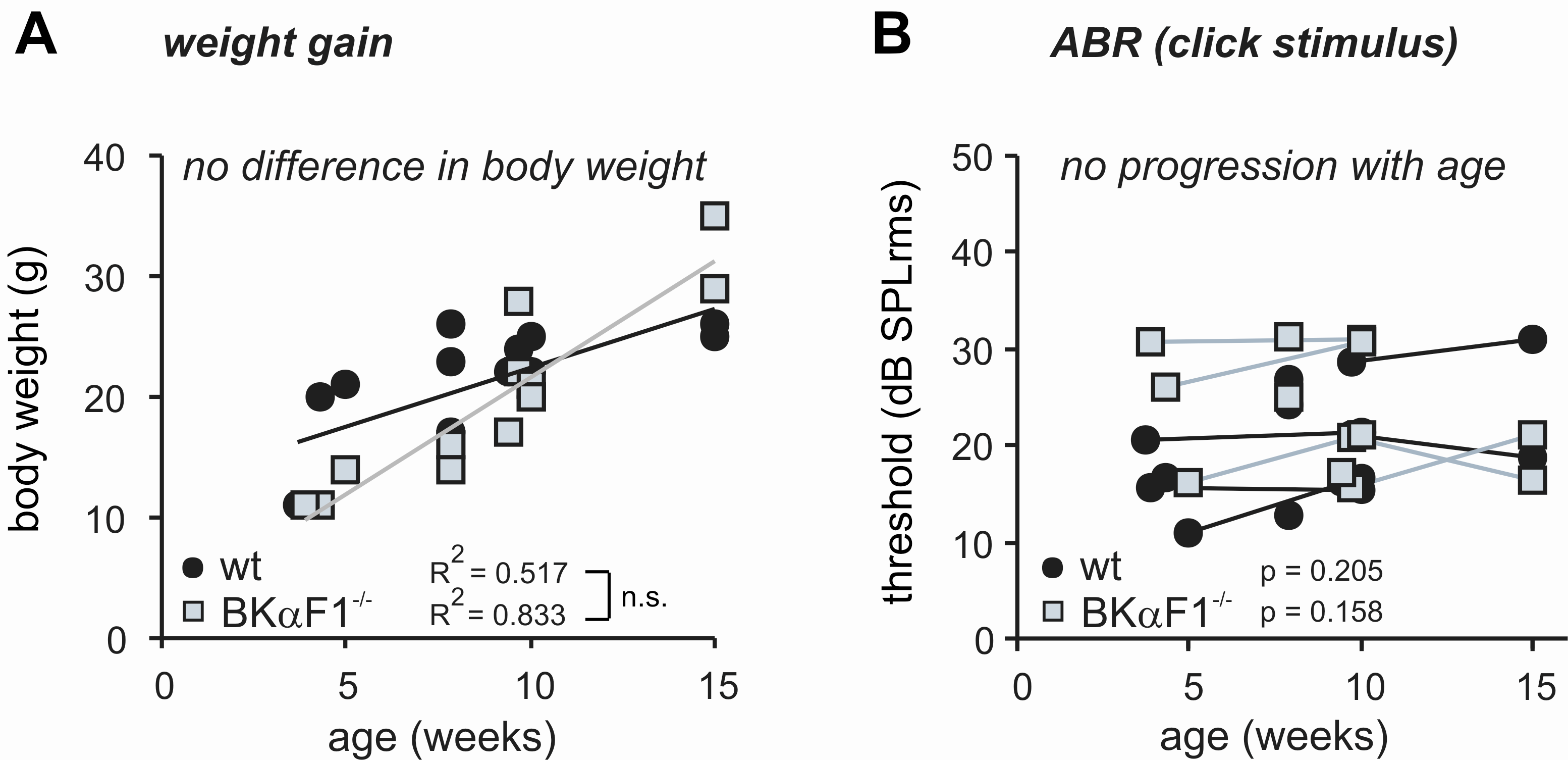

Supplement: Figure S2 — ABR hearing thresholds for WT (black circles) and BKα-/- mice (grey squares) for click stimuli did not change significantly with age. Animals measured on more than one time point are connected with lines. (TIF) [file pone.0081270.s002.tif]
